# Evaluation of the Effects of Extracorporeal Shockwave Therapy in Patients With Peripheral Arterial Disease: A Meta-Analysis of Randomized Control Trials

**DOI:** 10.7759/cureus.34729

**Published:** 2023-02-07

**Authors:** Zaid Munir, Muhammad Akash, FNU Jaiprada, Bilal Abu Tarboush, Osama Ijaz, Anan Bseiso, Sujith K Palleti, Adil Amin

**Affiliations:** 1 General Medicine, Withybush General Hospital, Cardiff, GBR; 2 Clinical Sciences, Mayo Hospital, King Edward Medical University, Lahore, PAK; 3 College of Medicine, Dow University of Health Sciences, Karachi, PAK; 4 Medicine, Universite Djillali Liabes Sidi Bel Abbes, Sidi Bel Abbès, DZA; 5 Internal Medicine, Services Institute of Medical Sciences, Lahore, PAK; 6 College of Medicine, Al-Quds University, Jerusalem, PSE; 7 College of Medicine, Hebron University, Hebron, PSE; 8 Nephrology, Loyola University Medical Center, Chicago, USA; 9 Cardiology, Pakistan Navy Station (PNS) Shifa, Karachi, PAK

**Keywords:** ankle brachial pressure index, clinical outcomes, meta-analysis, shock wave, peripheral arterial disease

## Abstract

The aim of this meta-analysis is to assess the efficacy of extracorporeal shockwave therapy (ESWT) in patients with peripheral arterial disease (PAD). This meta-analysis was performed according to Preferred Reporting Items for Systematic Reviews and Meta-Analyses (PRISMA) guidelines. A systematic search was conducted independently by two authors using PubMed, EMBASE, and Cochrane Central Register of Controlled Trials (CENTRAL) from inception to January 15, 2023. Primary clinical outcomes assessed in this meta-analysis were changes in maximum waking distance (MWD) and pain-free walking distance (PFWD) from baseline. Other outcomes assessed included change in ankle brachial pressure index (ABI) and degree of arterial stenosis. Four RCTs involving a total of 228 patients were included. Change of PFWD and MWD from baseline was significantly higher in patients randomized in the ESWT group as compared to the control group. No significant differences were reported between the two groups in terms of change in ABI from baseline. In conclusion, this meta-analysis of four randomized controlled trials found evidence that ESWT is an effective treatment for patients with PAD in terms of improving PFWD and MWD and reducing stenosis. However, there was no significant difference in the improvement of the ankle-brachial index between the study groups.

## Introduction and background

Peripheral arterial disease (PAD) is a condition caused by atherosclerosis that affects the non-intracranial and non-cardiac arteries [1]. It is characterized by damage to the innermost layer of the arteries, particularly the aorta and its branches. Risk factors for PAD include high blood pressure, high cholesterol levels, and smoking [2]. PAD can negatively impact both life expectancy and quality of life [3]. In recent years, the number of individuals living with PAD has increased, particularly in low and middle-income countries [4].

PAD is one of the leading causes of morbidity worldwide and can lead to prolonged hospital stays. It places a significant burden on the healthcare system [5]. The most common symptom of PAD is cramping leg pain that occurs when walking and is relieved by rest. Individuals with PAD often experience a poor quality of life [6]. Therefore, treatment aims to alleviate symptoms and improve quality of life. Treatment options include drugs, open bypass surgery, endovascular therapy, and exercise, with drugs and endovascular therapy being the most commonly used methods [7].

Recently, the National Institute for Health and Care Excellence (NICE) has recommended supervised exercise programs (SEP) as a first-line treatment for intermittent claudication due to convincing evidence of the clinical and symptomatic benefits [8]. Despite this evidence, SEP is not widely used, and patients have poor uptake and adherence rates [9]. Patients, vascular surgeons, and the healthcare system would likely be interested in an alternative noninvasive treatment option for PAD that is safe, well-tolerated, and demonstrates both clinical efficacy and cost-effectiveness [10].

Extracorporeal shockwave therapy (ESWT) is a new approach to the treatment of PAD. Shockwave is a transient acoustic pulse with a high peak pressure. Various energy frequencies and values are utilized for treating various diseases [11]. The shockwave system focuses on the gastrocnemius muscles in the lower leg during each PAD treatment session, which typically lasts for several minutes. It is commonly used in sports medicine and is now considered the primary treatment method for various sports-related conditions [12]. ESWT can increase blood flow in limbs by stimulating the formation of collateral circulation within them and delaying the process of arteriosclerosis in lower limbs, thus alleviating symptoms [13-14].

Several systematic reviews have been published emphasizing the use of extracorporeal shockwave therapy for urological and orthopedic conditions [15-16], and certain trials have been conducted to assess the efficacy of ESWT in the treatment of PAD. Despite this, its use in the management of PAD is not widely acknowledged by the medical community. This meta-analysis aims to summarize the available studies on the effect of ESWT on PAD to assess its safety and efficacy. The aim of this meta-analysis is to assess the efficacy of extracorporeal shockwave therapy in patients with PAD.

## Review

Methodology

This meta-analysis was performed according to Preferred Reporting Items for Systematic Reviews and Meta-Analyses (PRISMA) guidelines.

Search Strategy

A systematic search was conducted independently by two authors using PubMed, EMBASE, and Cochrane Central Register of Controlled Trials (CENTRAL) from their inception to January 15, 2023. Keywords used to search for relevant articles included "extracorporeal shockwave," "peripheral arterial disease," and "symptomatic management." Additionally, the reference lists of all selected articles were manually searched to identify relevant articles on the effectiveness of ESWT in patients with PAD.

Eligibility Criteria

Studies that assessed the safety and efficacy of extracorporeal shockwave therapy (ESWT) in patients diagnosed with PAD, including critical limb ischemia and intermittent claudication, were included. Studies published in languages other than English were excluded. We also excluded observational studies, case reports, case series, and review articles.

Study Selection

All articles were imported into the EndNote citation manager. After removing duplicate records, two authors independently performed initial screening using titles and abstracts to select articles compliant with the pre-defined eligibility criteria. The same two authors retrieved full texts of eligible studies to assess for inclusion and exclusion criteria. Any disagreement between the two authors was resolved through discussion and consensus.

Outcomes and Quality Assessment

Primary clinical outcomes assessed in this meta-analysis were changes in the maximum waking distance (MWD) and pain-free walking distance from baseline. Other outcomes assessed included change in ankle brachial pressure index (ABI) and degree of arterial stenosis.

The quality of all included studies was assessed using the Cochrane risk of bias assessment by two authors independently. Risk of bias ratings were assigned as follows: high risk of bias, moderate risk of bias, and low risk of bias. Any disagreement between two authors in the process of assessment of the risk of bias was resolved through discussion.

Data Extraction and Data Synthesis

Two researchers extracted the data using a pre-designed data extraction form, and one author entered the data into RevMan software (Review Manager, version 5.4.0; Nordic Cochrane Centre, Cochrane Collaboration) for analysis. Discrepancies between the two authors were resolved through consensus. Items extracted included the first author's name, publication year, number of participants, study duration, patients' characteristics (mean age and gender), and outcomes. Data analyses were performed using the Review Manager software. For continuous outcomes, we calculated means and their 95% confidence intervals (CIs). All reported P-values were obtained from two-sided tests, and values less than 0.05 were considered significant. Statistical heterogeneity was assessed using I-square statistics. An I-square value of 25% indicates mild, 50% indicates moderate, and 75% indicates high heterogeneity. Cochran-Q statistics were used to assess heterogeneity among the study results. When the p-value of the Cochran-Q statistics was less than 0.10, there was statistically significant heterogeneity among the study results.

Results

**Figure 1:**
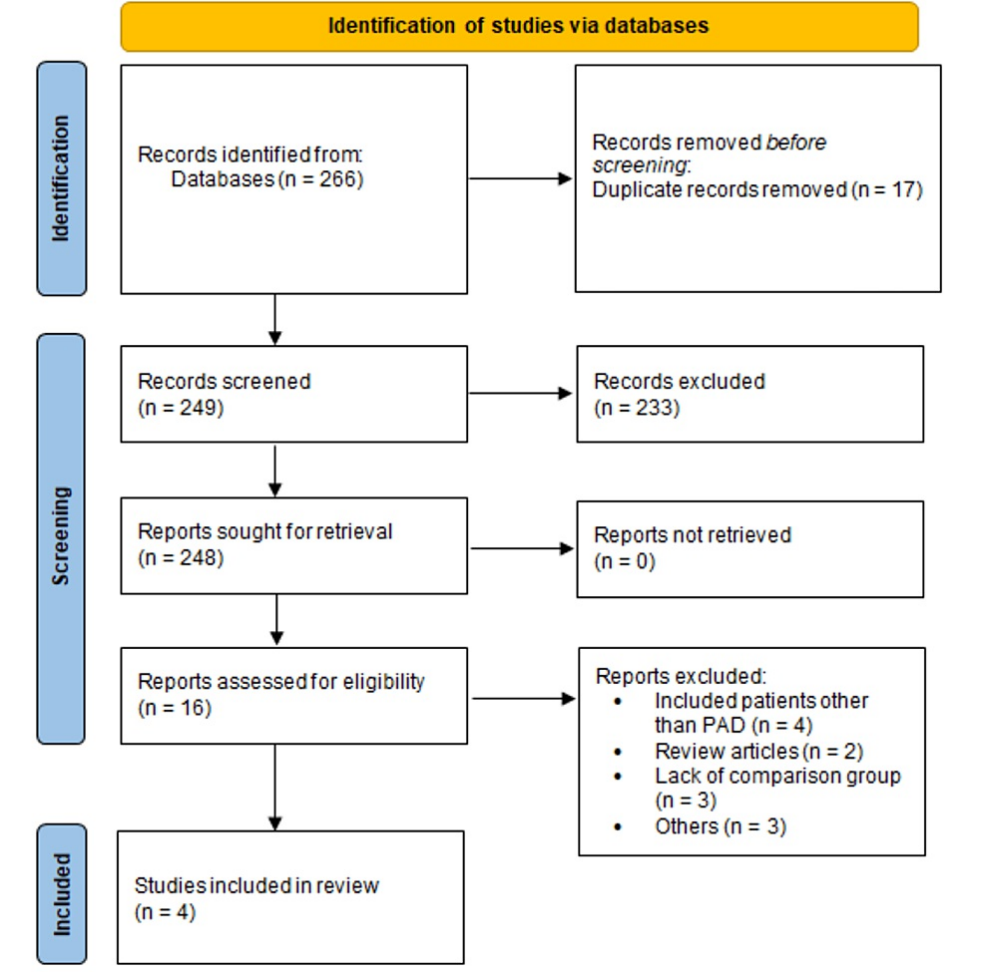
PRISMA flowchart of selection of studies PRISMA: Preferred Reporting Items for Systematic Reviews and Meta-Analyses

**Figure 2:**
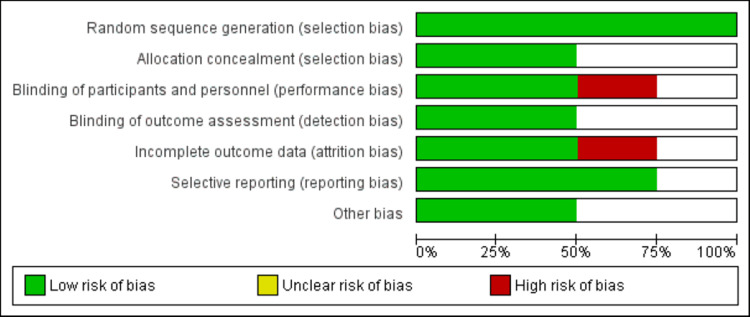
Risk of bias graph

Figure 1 shows the PRISMA flowchart of the selection of studies. A systematic search of the online databases yielded 266 articles. After removing duplicates, two authors independently screened the titles and abstracts of 249 studies to identify any potential article for inclusion. We retrieved the full texts of 16 studies and reviewed them for eligibility criteria. Finally, four RCTs involving a total of 228 patients were included in the meta-analysis. The age of participants ranged from 51.2 to 67.5 years. The sample size ranged from 22 to 138. The baseline characteristics of the included studies are shown in Table 1. The results of the risk of bias assessment are shown in Figure 2.

**Table 1:** Characteristics of included studies PAD: peripheral arterial disease; EWST: extracorporeal shockwave therapy

Author Name	Year of Publication	Participants	Groups	Sample Size	Follow-up	Mean age (Years)	Males (%)
Ali et al [17]	2022	Patients having intermittent claudication	ESWT	30	8 Weeks	51.2	NR
			Control	30			
Cai et al [18]	2021	Patients have lower limb intermittent claudication	ESWT	69	12 Weeks	NR	NR
			Control	69			
Ciccone et al [19]	2012	Patients with PAD Fontaine stages II–IV	ESWT	12	8 Weeks	67.5	86.40%
			Control	10			
Harwood et al [10]	2017	Patients have unilateral intermittent claudication	ESWT	15	12 Weeks	65.8	60%
			Control	15			

Outcomes

**Figure 3:**
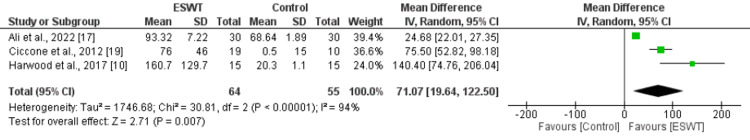
Effect of ESWT on change in PFWD Sources: References [10,17,19] EWST: extracorporeal shockwave therapy; PFWD: pain-free walking distance

Pain-free walking distance (PFWD):* *Three studies contained data for PWFD involving 119 patients with PAD. Change of PFWD from baseline was significantly higher in patients randomized in the ESWT group compared to the control group (MD: 71.17 meters, 95% CI: 19.64-122.50, p-value: 0.007) as shown in Figure 3. Significant heterogeneity was reported among the study results (I-square: 94%, p-value: 0.001).

**Figure 4:**
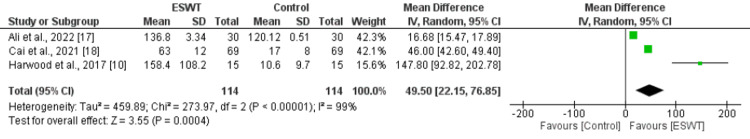
Effect of ESWT on change in MWD from baseline Sources: References [10,17-18] EWST: extracorporeal shockwave therapy; MWD: maximum walking distance

MWD:* *Three studies contained data for the MWD involving a total of 228 patients with PAD. Meta-analysis showed that the increase in MWD from baseline was significantly higher in patients randomized in the ESWT group compared to the control group (MD: 49.50 meters, 95% CI: 22.15-76.85, p-value<0.001) as shown in Figure 4. Significant heterogeneity was reported among the study results (I-square: 99%, p-value< 0.001).

**Figure 5:**
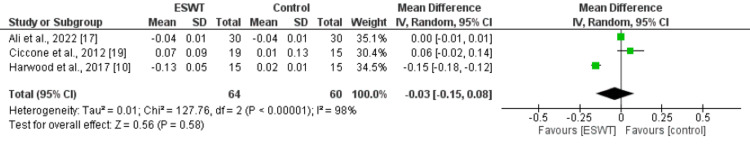
Effect of ESWT on change in ABI from baseline Sources: References [10,17,19] EWST: extracorporeal shockwave therapy; ABI: ankle-brachial index

ABI:* *Three studies compared changes in ABI from baseline between patients in two study groups. No significant difference was found between the two groups in a change of ABI from baseline (MD: -0.03, 95% CI: -0.15, 0.08, p-value: 0.57) as shown in Figure 5. Significant heterogeneity was reported among the study results (I-square: 98%, p-value < 0.001).

Degree of Arterial Stenosis

Ciccone et al. assessed the stenosis pre and post-intervention in two study groups [19]. The reduction in the mean degree of stenosis was significantly greater in patients receiving ESWT treatment compared with the control group (MD: -8.50, 95% CI: -13, -4, p-value <0.001). As only one study was involved in this outcome, we could not calculate heterogeneity.

Discussion

The purpose of this meta-analysis is to evaluate the effects of extracorporeal shockwave therapy (ESWT) on clinical outcomes in patients with peripheral artery disease (PAD). The data suggest that ESWT led to significant improvements in maximum walking distance (MWD) and pain-free walking distance (PFWD). However, there was no significant difference in the change in ankle-brachial index (ABI) score between the ESWT and control groups. In the study conducted by Serizawa et al. involving 12 patients receiving ESWT therapy, the MWD was significantly increased at four weeks and eight weeks from baseline [20]. Studies included in the meta-analysis that assessed PFWD and MWD as outcomes all recorded significant improvements after ESWT [10,17-19]. These improvements in MWD and PFWD are comparable to those observed after supervised exercise regimens and percutaneous transluminal angioplasty [21]. The meta-analysis also suggests that ESWT angiogenic response is potentially at a microvascular level and has no major effect on macrovascular circulation.

The improvements reported in MWD and PFWD in our meta-analysis did not correspond to any significant changes in ABI, showing that ESWT angiogenic response is potentially at a microvascular level and has no major effect on macrovascular circulation. The study conducted by Serizawa et al. reported no significant changes in ABI during the follow-up period in patients receiving ESWT [20].

Previous non-randomized studies have shown changes to the microcirculation in patients receiving ESWT [22], but the exact mechanism by which low-energy ESWT enhances blood flow in the microcirculation remains unknown [23]. Current clinical guidelines recommend supervised exercise as the first-line treatment for PAD after optimizing medical management and addressing modifiable risk factors. However, many patients with PAD are unwilling or unable to participate in supervised exercise therapy, so alternative treatments that are acceptable to patients would be a valuable addition to currently available therapies [24-25]. Thus efficient alternative treatments that are acceptable to patients will be a valuable addition to presently available therapies. Considering the benefits of ESWT, more prospective, multicenter trials need to be conducted, including a larger sample size, to explore the impact of ESWT on clinical benefits, including microcirculation, macrovascular circulation, and quality of life. It will help in the development of guidelines and recommendations for the management of PAD.

The results of this meta-analysis suggest that ESWT may be an effective treatment option for patients with PAD, but more research is needed to fully understand the long-term effects of ESWT, its cost-effectiveness in comparison to other treatment options, and its safety and side-effects profile. Further studies, including larger, multicenter randomized controlled trials and long-term follow-up studies, are needed to confirm the findings of the current literature and to explore the impact of ESWT on microcirculation, macrovascular circulation, and quality of life. Additionally, research is needed to investigate the mechanisms by which ESWT improves microcirculation in patients with PAD and to identify potential biomarkers that can be used to predict response to treatment.

Study Limitations

The limitations of this study related to the impact of ESWT on PAD include the small sample size of the studies included in the meta-analysis, which limits the generalizability of the results. Additionally, the low number of studies included in the analysis prevented the conduct of subgroup analysis, which would have provided insight into specific subpopulations that may benefit from ESWT. Furthermore, there was a high degree of heterogeneity among the study outcomes, which reduces the validity of the conclusions that can be drawn from the meta-analysis. This highlights the need for further research with larger sample sizes and more homogenous study populations to better understand the potential benefits of ESWT for patients with PAD.

## Conclusions

In conclusion, this meta-analysis of four randomized controlled trials found evidence that extracorporeal shockwave therapy (ESWT) is an effective treatment for patients with peripheral artery disease (PAD) in terms of improving pain-free walking distance (PFWD), maximum walking distance (MWD), and stenosis. However, there was no significant difference in the improvement of the ankle-brachial index between the study groups. Despite these promising results, further research is needed with larger sample sizes to fully assess the impact of ESWT on clinical outcomes and quality of life for patients with PAD. Additionally, it is essential to consider the cost-effectiveness of ESWT in comparison to other treatment options for PAD. Furthermore, future research should also focus on the long-term safety and efficacy of ESWT in treating PAD. ESWT may become a valuable alternative treatment option for individuals with PAD.
